# The Role of Tumor-Derived Exosomes (TEX) in Shaping Anti-Tumor Immune Competence

**DOI:** 10.3390/cells10113054

**Published:** 2021-11-06

**Authors:** Theresa L. Whiteside

**Affiliations:** Departments of Pathology, Immunology and Otolaryngology, University of Pittsburgh School of Medicine and UPMC Hillman Cancer Center, Pittsburgh, PA 15213, USA; whitesidetl@upmc.edu; Tel.: +1-(412)-624-0096; Fax: +1-(412)-624-0264

**Keywords:** tumor-derived exosomes (TEX), extracellular vesicles (EVs), adenosinergic pathway, immune suppression, tumor microenvironment (TME)

## Abstract

Emerging studies suggest that extracellular vesicles (EVs) mediating intercellular communication in the tumor microenvironment (TME) play a key role in driving cancer progression. Tumor-derived small EVs or exosomes (TEX) enriched in immunosuppressive proteins or in microRNAs targeting suppressive pathways in recipient cells contribute to reprogramming the TME into a cancer-promoting milieu. The adenosinergic pathway is an acknowledged major contributor to tumor-induced immune suppression. TEX carry the components of this pathway and utilize ATP to produce adenosine (ADO). TEX-associated ADO emerges as a key factor in the suppression of T cell responses to therapy. Here, the significance of the ADO pathway in TEX is discussed as a highly effective mechanism of cancer-driven immune cell suppression and of resistance to immune therapies.

## 1. Introduction

Immune suppression of the adaptive and innate anti-tumor response is an acknowledged contributor to cancer progression [1]. The recent success of immunotherapy with check point inhibitors (ICI) has shown that even a partial restoration of anti-tumor immune responses in patients with cancer leads to long-term survival in responders [2,3]. However, only a subset of cancer patients ranging from 20–50% (the percentage varies with the tumor type and clinical protocols used) of those treated are responders to ICI therapies [4,5]. The lack of response to immune therapy in so many cancer patients has stimulated an intense search for the potential cause of this diversity. Not surprisingly, examination of the tumor microenvironment (TME) has become the major focus of the search for molecular/genetic pathways that might underlie resistance of cancer patients to ICIs [6].

In the last two decades, an extensive volume of data has emerged that has illuminated an enormous complexity of the TME, its heterogeneity in different tumor types and its unique characteristics that shape tumor development and progression in every cancer patient. Features of the TME, such as the degree of infiltration with immune cells, immune cell localization, mutational tumor burden (TMB), loss of heterozygosity (LOH), cytokine/chemokine signatures and the presence in tumors of tertiary lymphoid structures (TLSs), have been interrogated and correlated with patients’ responses or lack of responses to the immune checkpoint blockade (ICB) [7,8,9]. Investigations of molecular pathways operating in the TME have resulted in the definition of prevalent molecular signatures for different tumor types [10]. Furthermore, the key role of intercellular communication within the TME as the major mechanism driving tumor progression has emerged, and understanding of the crosstalk between various immune and tissue cells has become the major goal of cancer research. For many decades, soluble factors, including cytokines and chemokines, have been held responsible for the regulation of the cellular milieu in healthy and pathological tissues. The recognition of extracellular vesicles, EVs, as participants of the intercellular communication network occurred only a few years ago [11]. Since then, EVs produced by the tumor and by immune as well as non-immune cells residing in the TME have become an object of intense interest, and numerous studies evaluating their role in shaping innate and adaptive immune responses in cancer are in progress.

## 2. Extracellular Vesicles in Cancer

Extracellular vesicles (EVs) are produced and released into the extracellular space by all cells. EVs are classified based on their biogenesis and heterogeneity in size as well as functions. The current nomenclature includes exosomes (30–150 nm), microvesicles (MVs; 150–1000 nm) and apoptotic bodies (>1000 nm). Exosomes are a subset of extracellular vesicles (EVs) that contain exomeres (<35 nm), small exosomes (Exo-S, 50–100 nm) and large exosomes (Exo-L, 100–150 nm) [12]. Although exosomes are heterogenous in size, they all share a common origin from multivesicular bodies (MVBs) in late endosomes [13]. This unique biogenesis in the endocytic cell compartment differentiates exosomes from MVs, which bud from the surface of parent cells, and from apoptotic bodies derived from cells undergoing apoptosis. When MVBs fuse with the plasma membrane, exosomes are released into the extracellular space. Due to their origin, exosomes carry endocytic markers, such as TSG101, ALIX, flotillin and others, but do not contain cytoplasmic proteins, such as calnexin or GRPp94, and the topography of exosome molecular surface resembles that of their parent cells. The molecular and genetic contents of exosomes also mimic those in parental cells. This similarity of molecular and genetic signatures of tumor-derived exosomes to parent tumor cells is the main reason for their potential status as a “liquid tumor biopsy” [14]. Tumor cells produce large numbers of exosomes, and plasma of cancer patients is enriched in tumor-derived exosomes called TEX [15]. Activated immune cells in the TME also avidly produce exosomes, which constitute a considerable proportion of the total EVs in plasma of cancer patients [15,16]. Through paracrine and juxtacrine interactions with TEX, immune cells in the TME are reprogrammed, and the exosomes these immune cells release in turn are equipped to promote tumor growth. This process of immune cell “corruption” or “subversion” by TEX is a part of the program orchestrated by the tumor and is aimed at changing the TME into one promoting tumor growth and suppressing anti-tumor functions of immune cells [17].

The result of tumor-driven reprogramming of immune cells in the TME is that TEX and immune cell-derived exosomes in plasma of cancer patients are enriched in immuno-suppressive proteins, and, upon co-incubation with primary normal immune cells or upon injection into experimental animals, these exosomes mediate immune suppression [18]. Although TEX carry tumor-associated antigens (TAAs) and thus could be immunogenic, TEX interactions with reprogrammed antigen-presenting cells (APCs) in the TME do not support antigen processing/presentation, which normally culminates in T cell responses [19]. Instead, T cells cross talking with TEX are suppressed or induced to acquire a suppressive phenotype (i.e., develop into Treg or myeloid-derived suppressor cells). Mechanistically, TEX-mediated immune suppression involves activation in recipient immune cells of numerous inhibitory pathways, leading to a loss of anti-tumor functions [20]. Suppressive activities of TEX appear to be the major element of negative regulation that prevails in the TME. To illustrate how tumors utilize TEX to promote and maintain immune suppression in the TME, we will focus on activation of the cAMP-mediated adenosine synthesizing pathway, one of the major regulatory pathways responsible for immune suppression.

## 3. The Adenosinergic Pathway in TEX

Exosomes, serving as a communication system between cells, deliver their cargos to recipient cells and profoundly alter the phenotype and function of exosome-receiving cells. Tumor cells release large numbers of exosomes into body fluids (e.g., ~10^12^ TEX/mL) [21], which carry and simultaneously deliver multiple inhibitory signals to recipient cells. Ultimately, this leads to T-cell apoptosis [22]. In contrast, exosomes derived from non-malignant cells carry an excess of stimulatory signaling proteins and tend to activate T-cells [22]. Thus, TEX emerge as a major immunoregulatory system which tumors exploit to defend themselves against immunological attack.

The adenosinergic pathway is acknowledged as a major contributor of tumor-induced suppression of immune cells and a promoter of tumor growth and metastasis in various cancer types [23]. In cancer patients, TEX-associated adenosine (ADO) mediates suppression of T cell responses to immunotherapy [24]. Both canonical (extracellular) and non-canonical (intracellular) adenosinergic pathways lead to the production of adenosine (ADO), which signals via four adenosine receptors (ADORs: A_1_R, A_2A_R, A_2B_R and A_3_R), also known as purinergic type 1 receptors (P1Rs). The P1Rs are widely distributed among diverse cell types. ADO mediates pro-tumor activities by inducing tumor cell proliferation, angiogenesis, chemoresistance, and migration/invasion by tumor cells (reviewed in ref [25]). ADO, via G-protein coupled receptors, also inhibits proliferation and other functions of both CD4^+^ T helper cells and CD8^+^ cytotoxic T cells, favoriting tumor escape from the host immune system [25]. This suggests that ADO is a *bona fide* therapeutic target and *a major immune checkpoint in cancer immunology*. First, TEX carry at least two of the most important ectoenzymes that produce ADO, namely CD39 (converts ATP to ADP and ADP to AMP) and CD73 (converts AMP to ADO). We reported that TEX readily metabolize ATP to ADO and that of 20 different purines, ADO is the most abundant in TEX. Thus, TEX are not only carriers of intraluminal ADO, but they actively produce ADO [26]. Since the half-life of free ADO in human blood is only approximately one second, our finding that exosomes are a source of extracellular ADO in plasma represents a paradigm-shifting concept.

ADO packaged in TEX is protected from rapid uptake and metabolism by red blood cells and can, therefore, be delivered directly to target cells, where it can exert biological effects. In cancer patients’ plasma, TEX carrying intra-vesicular ADO to recipient immune and tumor cells emerge as a major source of immunosuppressive ADO, as well as of ADO mediating pro-tumor activities. We showed that TEX produced by head and neck squamous cell carcinoma (HNSCC) or melanoma cells carry CD39 and CD73 on their surface and exhibit potent ATP-AMP phosphohydrolytic activities [22,27]. These TEX produced by multiple myeloma cells were reported to be equipped with CD39/CD73 and with the enzymes that generate ADO via the non-canonical pathway (NAD^+^/CD38/CD203a/CD73), and thus were able to generate ADO utilizing both the canonical and non-canonical pathways [24]. ADO produced by TEX was shown to inhibit T-cell activation and proliferation through A_2A_Rs [28]. ADO production by tumor cells and the tumor-promoting effects of ADO appear to be universal attributes of malignancy in hematologic as well as solid tumors [29].

**Figure 1:**
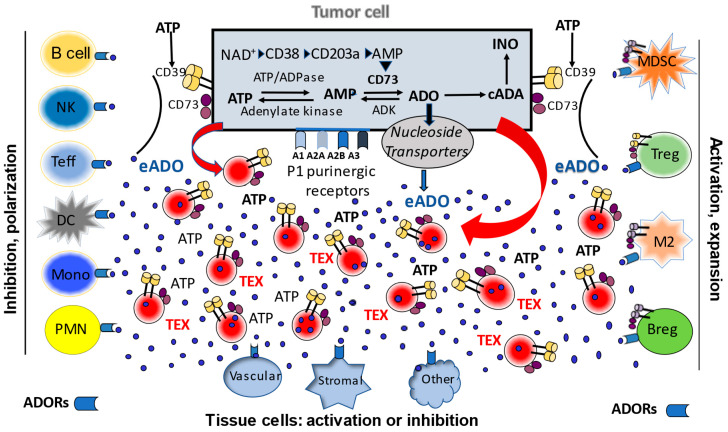
A schematic view of intracellular and extracellular adenosinergic pathways in the tumor microenvironment (TME). Tumor cells operate a canonical ATP/CD39/CD73 pathway and non-canonical NAD^+^CD38/CD203 pathway. The canonical pathway involves sequential hydrolysis of ATP to ADP to AMP mediated by CD39 and CD73 to produce ADO. The non-canonical pathway involves the use of NAD^+^ as a substrate by CD38 to generate ADP-ribose, which is processed by CD203a to AMP and then by CD73 to ADO. Once generated, ADO is either metabolized to inosine by adenosine deaminase (cADA) or transported to the extracellular space by nucleoside transporters. Extracelular (e)ADO interacts with ADORs (type 1 purinergic (P1) receptors) broadly expressed on all cells in the TME, including tumor cells. ADO signals may be inhibitory or stimulatory, depending on the type of adenosine receptors (ADORs: A1, A2a, A2b, A3). Tumor cells produce and release extracellular vesicles called exosomes (TEX). These are released into extracellular space in large numbers and carry surface ectonucleotidases CD39/CD73. In the presence of ATP excess in the TME, TEX produce ADO. They also carry intraluminal ADO and deliver it to recipient cells upon uptake into cytosol. TEX also deliver biologically active CD39/CD73 to recipient cells, providing them with the enzymatic capability to metabolize ATP into ADO. TEX emerge as a major driver of ADO-mediated signaling in the TME.

In addition to directly delivering ADO to recipient cells, TEX upregulate ADO production in these cells. For example, prostate cancer-derived exosomes were reported to induce CD73 expression in dendritic cells (DC), which led to an inhibition of tumor necrosis factor-alpha (TNF-α) and IL-12 production by T lymphocytes in an ATP-dependent manner [19]. TEX released from HNSCC cells carrying CD39 and CD73 increased ADO production in regulatory T cells (Treg) [30]. Autocrine effects of TEX on tumor cell growth, tumor resistance to chemotherapies and establishment of metastases have been reported in numerous in vitro and in vivo studies in animal tumor models [25,31,32]. Via juxtacrine or paracrine signaling in the tumor microenvironment (TME), TEX are known to alter functions of mesenchymal stem cells, fibroblasts and endothelial cells [17]. In aggregate, TEX-mediated changes in recipient cells are the result of receptor-ligand interactions on the cell surface and/or up-take by recipient cells of micro-RNAs (miRs) carried by TEX [29,33,34]. Further, TEX enriched in multiple angiogenic proteins can directly promote migration of endothelial cells, vessel sprouting, tubule formation and growth [35,36]. More recent data suggest the presence of a link between angiogenesis and TEX-associated components of the adenosinergic pathway, including ADO [26,35,37,38]. Figure 1 illustrates the potential role of TEX in mediating extracellular and intracellular adenosinergic pathways in the tumor microenvironment. 

## 4. Significance of the TEX-Mediated Adenosinergic Pathway

The available data place TEX in a new role as a highly effective mechanism of cancer-driven immune suppression and pro-tumor activities that involves the ADO pathway. Tumor-induced immune suppression and pro-tumor activities mediated by TEX are key components of the intricate program tumor cells have developed to favor their survival and resistance to anti-cancer therapies, including therapy with ICIs. TEX represent a highly versatile version of the communication system used by normal cells that tumors have hijacked and adapted to promote tumor progression [20]. TEX circulate freely, delivering pro-tumor and anti-immune response signals to a broad variety of cells, and represent a major barrier to anti-tumor immune therapies as well as chemotherapies [16]. TEX carry components of various molecular pathways tumors engage for self-preservation, e.g., the TGF-β or FAS/FasL pathways [39]. Among these pathways, the adenosinergic pathway appears to play a prominent role mediated by ubiquitous ADO-carrying and ADO-producing TEX in body fluids.

Since the role of ADO as a contributor to tumor progression has been recognized in recent years, numerous pre-clinical and clinical studies utilizing pharmacologic inhibitors, siRNA or antibodies specific for the components of the adenosine pathway and antagonists of adenosine receptors have been conducted (reviewed in [40,41,42]). Pre-clinical studies in various in vitro and in vivo tumor models have shown efficacy and are currently entering the clinical arena [25,43,44]. While anti-ADO therapies alone or in combination with ICIs are being tested in phase I clinical trials, yet another mechanism of tumor-driven immune suppression and promotion of tumor growth that involves ADO-carrying exosomes has emerged, creating concerns about the therapeutic efficacy of current anti-ADO strategies. Thus, the TEX-driven mechanism of cancer promotion represents yet another hurdle to be overcome in eliminating tumor-induced immune suppression. The hypothesis underlying the significance of ADO-carrying TEX in cancer is that excessive numbers of ADO-producing TEX in plasma of cancer patients predict poor prognosis, and that patients with TEX expressing high “adenosinergic activity” would benefit from anti-adenosinergic therapy and should not receive therapies that increase the numbers of TEX carrying ADO. Although therapies targeting the ADO pathway operating in the tumor milieu are currently available in the clinic [43], they do not consider and, therefore, do not target the TEX-mediated effects. While there is a consensus that high “adenosinergic activity” is undesirable for immunotherapy of cancer, it remains unclear how much of this activity can be ascribed to TEX. Methodologies for the isolation of TEX from body fluids are just emerging, and efforts are being made to estimate the contribution of TEX to overall “adenosinergic activity” in patients with cancer. These efforts are driven by a suspicion that TEX-driven effects are underestimated and might account for the limited anti-tumor effects of current immunotherapy. The understanding of mechanisms underlying the activity of TEX-delivered ADO and developing strategies that inhibit this activity in vivo is critically important and represents a novel approach to the adequate control of ADO-induced immune suppression and tumor growth promotion. To this end, studies of the role of the adenosinergic pathway in TEX are now in progress in our laboratory and are expected to provide new insights into the mechanisms of tumor progression and metastasis and to reveal novel strategies for cancer immunotherapy alone or in combination with chemo/radiotherapy.
